# Arterial thoracic outlet syndrome due to a first-rib anomaly causing brachial artery embolic occlusion: a case report

**DOI:** 10.1016/j.radcr.2026.02.020

**Published:** 2026-03-14

**Authors:** Nahiro Yan, Fumie Sugihara, Koichi Akutsu, Taiga Matsumoto, Tatsuo Ueda, Hidemasa Saito, Ryutaro Fujitsuna, Sayaka Shirai, Shiori Shimizu, Hiromitsu Hayashi, Shin-Ichiro Kumita

**Affiliations:** a Department of Radiology, Nippon Medical School, Tokyo, Japan; b Department of Cardiovascular Medicine, Nippon Medical School, Tokyo, Japan

**Keywords:** Arterial thoracic outlet syndrome, First-rib malformation, Arterial stenosis, Three-dimensional reconstruction, Volume rendering

## Abstract

Arterial thoracic outlet syndrome is a rare condition caused by compression of the subclavian artery at the thoracic outlet, which often leads to delayed diagnosis and potentially severe outcomes such as upper limb necrosis. We report a case of a man in his 20s who presented with coldness in the right fingers, exacerbated by cold environments. Chest radiography revealed abnormal bone structures, and contrast-enhanced computed tomography confirmed stenosis and post-stenotic dilation of the right subclavian artery, thrombus formation, and occlusion of the brachial artery. Although bilateral first-rib anomalies were present, three-dimensional volume-rendered imaging identified focal arterial compression on the right side due to abnormal fusion of the first and second ribs, leading to a definitive diagnosis of arterial thoracic outlet syndrome. This case highlights that reliance on routine two-dimensional imaging alone may contribute to diagnostic delay, whereas selective use of three-dimensional reconstruction can provide critical anatomical insight, enabling accurate diagnosis and appropriate management in patients with complex thoracic outlet anatomy.

## Introduction

Arterial thoracic outlet syndrome (aTOS) is a rare disorder caused by compression of the subclavian artery at the thoracic outlet, often due to congenital bony abnormalities such as cervical ribs or an abnormal first rib [1]. In addition to its infrequency, the symptoms are often nonspecific, and diagnosis is often delayed [2]. However, aTOS can result in serious outcomes such as arterial stenosis, thrombus formation, embolism, and even necrosis due to ischemia of the upper extremities. Therefore, radiologists should be aware of this condition and perform accurate diagnostic imaging [2,3]. In this report, we describe a case of aTOS caused by a first-rib malformation that resulted in occlusion of the brachial artery.

## Case report

**Fig. 1:**
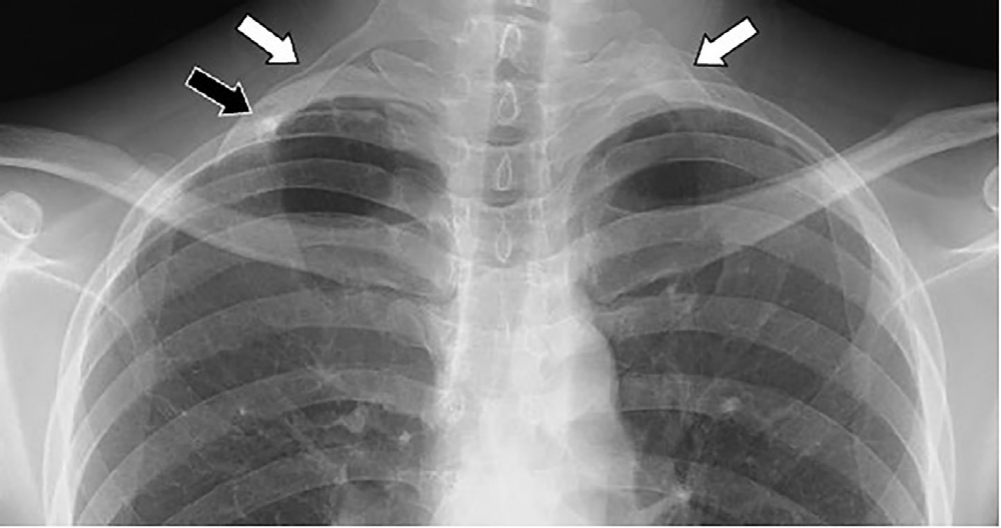
Chest radiography. Bilateral abnormal rib-like bony structures resembling cervical ribs are observed (white arrow). On the right side, focal osteosclerosis is noted at the site of overlap with a caudal bone (black arrow).

**Fig. 2:**
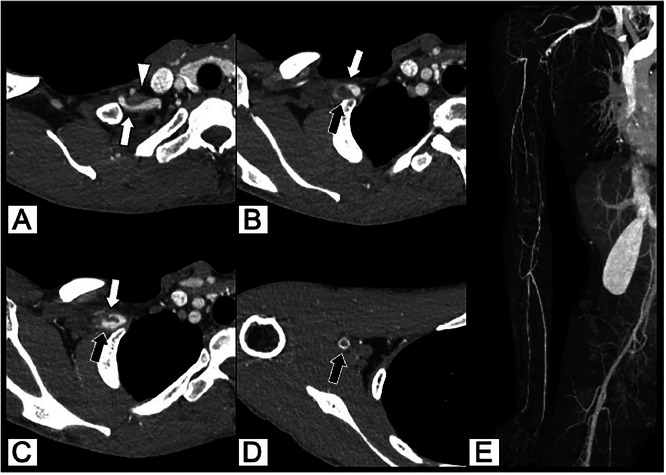
Contrast-enhanced computed tomography. (A) A stenosis is observed in the right subclavian artery (white arrow) that appears to be caused by compression of the anterior scalene muscle (arrowhead). (B-D) Post-stenotic dilatation is observed (white arrow), and a contrast filling defect suggestive of a thrombus is observed in the lumen (black arrow). (E) In the maximum-intensity projection three-dimensional reconstruction image, vascular occlusion is observed from the distal part of the right axillary artery to the brachial artery. Collateral circulation is well developed in the surrounding area, and blood flow to the forearm is maintained via collateral circulation.

**Fig. 3:**
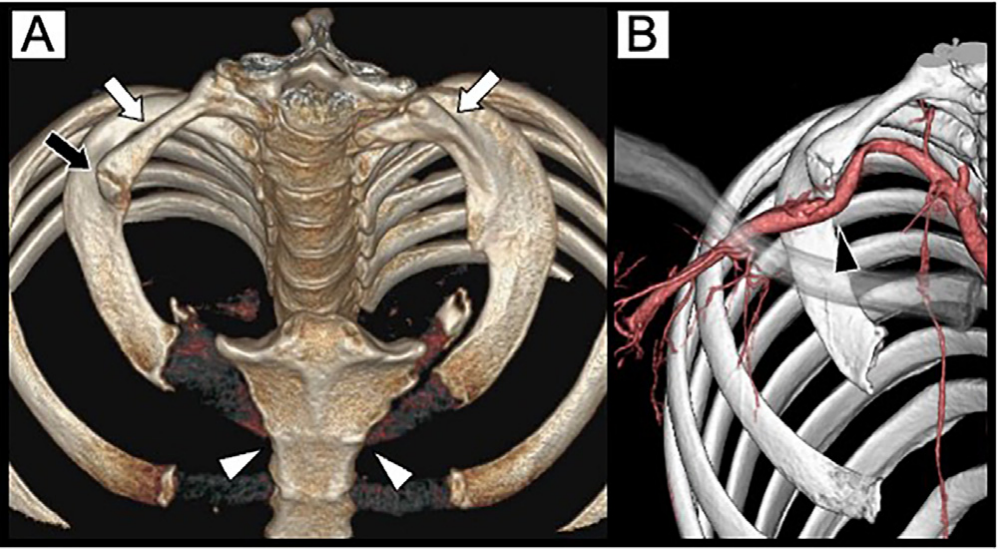
Volume-rendered three-dimensional reconstruction from the computed tomography image. (A) Abnormal bone structures resembling cervical ribs are observed on both sides (white arrows). These do not attach directly to the sternum and, on the right side, are fused with the caudal rib in a pseudoarthrosis-like structure (black arrow). The caudal ribs attach to the second sternal notch (white arrowheads). (B) The right subclavian artery is strongly compressed by the pseudoarthrosis-like structure (black arrowhead).

A man in his 20s presented with a chief complaint of coldness in his right forearm and fingers that had been worsening during exposure to cold environments during work for approximately 6 months. He was employed in transportation services. He possessed no relevant medical or smoking history. His blood pressure was 89/63 mmHg on the right side and 110/53 mmHg on the left side, with the right upper extremity being lower than the left upper extremity. Electrocardiography revealed sinus rhythm. Myeloperoxidase-antineutrophil cytoplasmic antibody, proteinase 3-antineutrophil cytoplasmic antibody, and antinuclear antibody were negative, and C-reactive protein as well as erythrocyte sedimentation rate were within normal ranges. Chest radiography revealed bilateral abnormal cervical rib-like bones. On the right side, osteosclerosis was observed in the area overlapping the caudal bone (Fig. 1). Contrast-enhanced computed tomography (CT) revealed stenosis and post-stenotic dilatation of the right subclavian artery, with a contrast defect suggestive of a thrombus in the dilated segment. A thrombus with well-developed collateral circulation in the surrounding area occluded the portion of the right axillary artery distal to the brachial artery. Blood flow to the forearm was maintained through collateral circulation (Fig. 2). Stenosis of the subclavian artery appeared to be caused by compression of the anterior scalene muscle; however, the cause was unclear based on the axial images alone. Therefore, we created a three-dimensional (3D) reconstruction using volume rendering (VR). This revealed that the bilateral cervical rib-like abnormalities were not directly attached to the sternum but were fused with the caudal rib in a pseudoarticular-like structure on the right side. The caudal rib was considered the second rib, as it was attached to the second costal notch of the sternum. Therefore, the abnormal bone was identified as an abnormal first rib. The abnormal first and second ribs were fused, forming a pseudoarthrosis-like structure that severely compressed the subclavian artery (Fig. 3). Additionally, an abnormal attachment of the anterior scalene muscle was observed. Based on these imaging findings, aTOS was diagnosed. Owing to marked venous enhancement and contrast-related artifacts caused by left-sided contrast injection, detailed evaluation of the left subclavian artery and vein was not feasible.

**Fig. 4:**
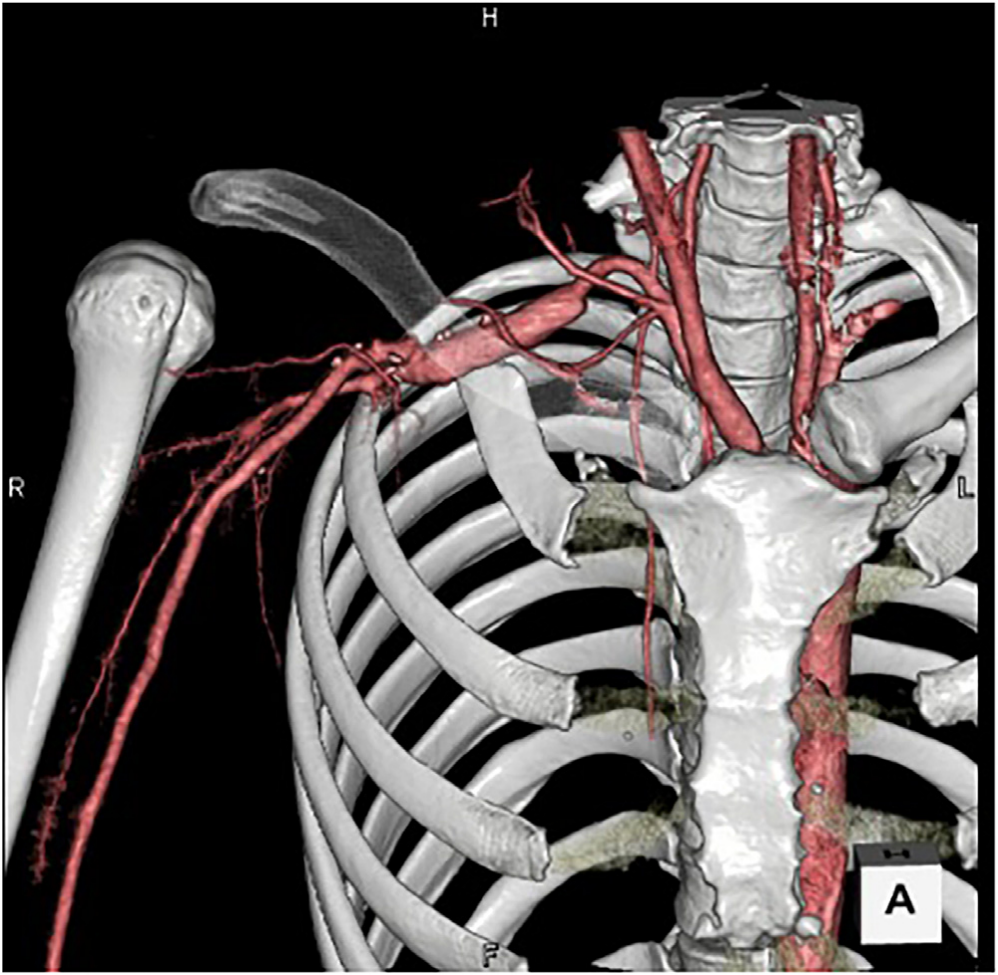
Volume-rendered three-dimensional reconstruction from postoperative contrast-enhanced computed tomography angiography demonstrating restored arterial flow and patency of the reconstructed right subclavian artery following graft interposition.

The patient was referred to a hospital specializing in thoracic outlet syndrome (TOS), where he underwent first and second rib resection, right subclavian artery replacement, and upper-arm artery bypass surgery using an autologous vein graft to restore blood flow. Postoperative 3D CT angiography demonstrated improved blood flow in the reconstructed right subclavian artery (Fig. 4). The patient was discharged 3 weeks after surgery, with complete resolution of symptoms.

## Discussion

In aTOS, the subclavian artery is compressed at the thoracic outlet. aTOS is caused by the bones, oblique muscles, and ligaments, among other factors. The causes of aTOS are mostly considered skeletal, with congenital bone abnormalities such as cervical ribs, first-rib anomalies, and elongated C7 transverse processes being common. Other causes include fractures of the clavicle or ribs. Cervical rib malformations are reported to be 3-4 times more common than first-rib malformations [1,3,4]. aTOS accounts for approximately 1% of all TOS cases. It is rare compared to neurogenic TOS (nTOS), in which the brachial plexus is compressed, and venous TOS (vTOS), in which the subclavian vein is compressed [3,5]. There have also been reports of individual patients presenting with more than 1 subtype of TOS [6]. The diagnosis of aTOS is often delayed due to its rarity and the wide range of nonspecific clinical manifestations, including claudication, pallor, coldness, and sensory abnormalities in the upper extremities. However, thrombi formed in the subclavian artery can embolize to peripheral arteries, resulting in ischemia of the upper extremity, with a serious outcome of amputation due to necrosis of the upper extremity in the worst cases [2,3].

As most patients do not experience constant arterial compression, there is no significant difference in the blood pressure measurements. However, a difference ≥20 mmHg in systolic blood pressure is considered significant [3]. In the present case, in addition to the right brachial artery not being palpable, systolic blood pressure was 21 mmHg lower in the symptomatic right upper extremity than in the left upper extremity. These findings may serve as indicators of arterial blood flow disorders of the upper limbs.

When aTOS is suspected, it is useful first to obtain plain radiographs of the cervical spine and chest to exclude bony abnormalities, because as mentioned above, the cause is often bony [1,3]. In the present case, only a plain chest radiograph was obtained, and bony abnormalities were identified within the scope of this radiograph.

For further examination, CT angiography (CTA) may be the most useful, particularly for understanding the relationship between the vasculature and bone. aTOS manifests as narrowing or post-stenotic dilation of the subclavian artery, thrombus formation, peripheral arterial occlusion, and development of collateral circulation, and CTA can accurately detect these vascular changes [3,7]. In the present case, the stenosis, post-stenotic dilatation of the right subclavian artery, thrombus within the dilated segment, occlusion of the right brachial artery, and collateral circulation were clearly visualized.

Although CTA is useful in diagnosing aTOS, two-dimensional (2D) cross-sectional images alone possess limitations in providing a 3D understanding of how bone abnormalities affect blood vessels. According to the literature, the VR method in 3D reconstruction exhibits higher sensitivity and specificity for detecting subclavian artery stenosis than that of 2D cross-sectional images and is particularly useful for intuitively understanding complex bone-muscle-vessel relationships (with 95% sensitivity and 100% specificity) [8]. In the present case, 2D cross-sectional images obtained immediately after the examination did not identify any compressive structures. However, when additional 3D reconstruction images were created, the compression of the subclavian artery due to abnormal fusion of the first and second ribs could be visualized, leading to a definitive diagnosis of aTOS.

Additionally, TOS imaging is often performed with the patient's arms in the adducted and abducted positions to capture vascular stenosis [9]. In the present case, with the upper extremities adducted, subclavian artery stenosis, a thrombus in the lumen, and peripheral emboli were evident, providing a definitive diagnosis. However, in patients without thrombi in the stenotic area or peripheral emboli, the ability to detect vascular stenosis affects diagnosis. Therefore, performing CTA, including positional changes, in patients suspected of experiencing aTOS may lead to a more accurate diagnosis.

Ultrasonography is a non-invasive, simple, and flexible imaging technique that allows for more adaptable positional changes, such as abduction of the upper extremity. However, it is difficult to observe the location of subclavian artery compression directly, and it may not be possible to identify other symptomatic lesions such as tumors more centrally. Therefore, ultrasonography alone cannot be used for adequate evaluation [5,9].

Magnetic resonance angiography involves no radiation exposure, is suitable for evaluating soft tissues and neurovascular bundles, and is excellent at depicting anatomical structures. However, the examination time is long (approximately 40 min) and is not suitable for symptomatic patients or those with claustrophobia. Additionally, some patients possess devices that preclude the performance of magnetic resonance imaging [2].

Angiography has historically been used to evaluate blood vessels; however, it is an invasive procedure that makes it difficult to visualize the bones and soft tissues that narrow the vessels. Therefore, its use has become limited primarily to the detection of aTOS complications such as thrombosis and embolism and the evaluation of peripheral circulation, particularly in the hands and fingers [2].

In such cases where nonspecific upper limb coldness and blood pressure differences are present, the following differential diagnoses should be considered. In the differential diagnosis of TOS, aside from nTOS and vTOS, attention should be paid to other vascular disorders, including Raynaud's phenomenon in collagen vascular disease, vasculitis such as Buerger's disease, arterial dissection, and cardiac-origin peripheral embolism with an embolic source [2,3]. Sensory abnormalities and muscle weakness are the primary clinical features of nTOS, and imaging studies often do not reveal clear vascular stenosis or emboli. In contrast, vTOS presents with swelling, edema, and dilation of the upper limb veins, and CT imaging may reveal narrowing or thrombus formation in the subclavian vein [1,9]. In the present case, the imaging findings demonstrated arterial narrowing, thrombus formation, and peripheral arterial occlusion, leading to a definitive diagnosis of aTOS.

Management of aTOS depends on symptom severity and arterial damage. Conservative treatment may be considered in asymptomatic patients, but is generally insufficient once fixed arterial compression is present [2,3]. Endovascular treatment can be used as an adjunct or bridging therapy; however, its durability is limited without surgical decompression because the underlying compression remains unresolved [3,10]. Surgical decompression remains the definitive treatment, and arterial reconstruction is required when arterial injury or distal embolization is present [2,3,10]. In the present case, brachial artery occlusion corresponded to stage III, for which surgical decompression with revascularization was indicated.

In contemporary radiology practice, increasing imaging volume often leads to image interpretation based predominantly on routine 2D images, while 3D reconstruction may be omitted due to time constraints.

In the present case, reliance on 2D images alone delayed recognition of the underlying compressive anatomy. 3D VR reconstruction was essential for visualizing the relationship between the anomalous first rib and the subclavian artery, enabling accurate diagnosis and appropriate surgical planning. This case highlights that selective use of 3D imaging can help prevent diagnostic delay in patients with complex thoracic outlet anatomy.

## Conclusions

This case demonstrates unilateral aTOS with a distal embolic complication despite bilateral first-rib anomalies and highlights the value of selective 3D CT imaging in identifying the responsible anatomy and preventing diagnostic delay.

## Patient consent

Informed consent was obtained from the patient for publication of the report and associated images.

## Appendix Supplementary materials

Image, application 1

## References

[1] Sanders R.J., Hammond S.L., Rao NM. (2007). Diagnosis of thoracic outlet syndrome. J Vasc Surg.

[2] Nguyen L.L., Soo Hoo AJ. (2021). Evaluation and management of arterial thoracic outlet syndrome. Thorac Surg Clin.

[3] Daniels B., Michaud L., Sease F Jr, Cassas K.J., Gray B.H. (2014). Arterial thoracic outlet syndrome. Curr Sports Med Rep.

[4] Sanders R.J., Haug C. (1991). Review of arterial thoracic outlet syndrome with a report of five new instances. Surg Gynecol Obstet.

[5] Demondion X., Herbinet P., Van Sint Jan S., Boutry N., Chantelot C., Cotten A. (2006). Imaging assessment of thoracic outlet syndrome. Radiographics.

[6] Lam T.Q., Nguyen A.D., Tran T.M., Van Hoang D., Quach TH. (2024). A rare case of overlapping thoracic outlet syndrome attributed to an anatomical variation in the anterior scalene muscle: diagnostic challenges and treatment approaches. Radiol Case Rep.

[7] Criado E., Berguer R., Greenfield L. (2010). The spectrum of arterial compression at the thoracic outlet. J Vasc Surg.

[8] Remy-Jardin M., Remy J., Masson P., Bonnel F., Debatselier P., Vinckier L. (2000). CT angiography of thoracic outlet syndrome: evaluation of imaging protocols for the detection of arterial stenosis. J Comput Assist Tomogr.

[9] Raptis C.A., Sridhar S., Thompson R.W., Fowler K.J., Bhalla S. (2016). Imaging of the patient with thoracic outlet syndrome. RadioGraphics.

[10] Potluri V.K., Li R.D., Crisostomo P., Bechara CF. (2024). A review of arterial thoracic outlet syndrome. Semin Vasc Surg.

